# Diverging cognitive benefits from education between rural and urban middle-aged and older adults in the USA

**DOI:** 10.1192/bjo.2025.45

**Published:** 2025-04-17

**Authors:** Roger Wong, Amer Mansour

**Affiliations:** Department of Public Health and Preventive Medicine, Norton College of Medicine, SUNY Upstate Medical University, Syracuse, NY, USA; Department of Geriatrics, Norton College of Medicine, SUNY Upstate Medical University, Syracuse, NY, USA; Norton College of Medicine, SUNY Upstate Medical University, Syracuse, NY, USA

**Keywords:** Cognitive decline, education, older adult, rural, urban

## Abstract

**Background:**

Subjective cognitive decline (SCD) is defined as self-reported increase in confusion or memory loss. There is limited research on the interplay between rural–urban residence and education on SCD.

**Aims:**

Examine rural–urban differences in SCD, and whether education moderates this relationship.

**Method:**

Respondents aged ≥45 years were queried about SCD in the 2022 Behavioral Risk Factor Surveillance System data, creating a sample size of 63 890. A logistic regression analysed the association between rural–urban residence and SCD, and moderation was tested by an interaction with education.

**Results:**

SCD was more common among rural (12.0%) compared with urban (10.7%) residents. Rural residence was associated with 9% significantly higher odds of SCD compared with urban residence after adjusting for sociodemographic and health covariates (adjusted odds ratio (aOR) = 1.09, *P* = 0.01). There was a negative relationship between education level and SCD, including the association of college degree with 15% lower odds of SCD compared with less than high school degree (aOR = 0.85, *P* < 0.01). Education was a significant moderator, with higher education associated with lower odds of SCD for urban, but not rural, residents.

**Conclusions:**

Rural setting and lower education were associated with higher odds of SCD, but higher education was protective for only urban residents. These results indicate that higher education may be a gateway for more opportunities and resources in urban settings, with cascading impacts on cognition. Future research should examine reasons for the diverging cognitive benefits from education depending on rural–urban residence.

Subjective cognitive decline (SCD) is a self-experienced decline in cognitive ability with limited or no impairment of performance on clinical standardised cognitive tests.^1^ One study on the epidemiology of SCD estimated its prevalence to be around 24% in patients above the age of 60 years.^2^ Research has identified a potential association between SCD and future cognitive impairment, with one systematic review reporting a 2.2-fold increase in risk of dementia for patients who suffer from SCD when compared with normal ageing.^3^ Similarly, a cohort study of nearly 560 000 patients above the age of 66 years noted a dementia incidence of 8.59 per 1000 person-years in patients with SCD compared with 5.66 in those without SCD.^4^ SCD, therefore, represents an important early indicator of dementia, which currently has limited strategies for management or prevention.

## Environment and cognition

Prior research has linked environmental and geographical factors to cognitive health. For example, one study examined how neighbourhood physical disorder and social cohesion were associated with dementia risk, using 9 years of data from a national sample of US Medicare beneficiaries.^5^ One of the key findings was that high physical disorder (litter, graffiti, housing/storefront vacancies) was associated with an 11% significantly higher dementia risk, which is probably explained by unfavourable settings that limit the ability to engage in exercise and other health behaviours. In rural ageing research, a 2018 paper using Health and Retirement Study (HRS) data found there was a 79% increased dementia risk, which may be explained by well-documented gaps in a variety of medical, public health and social services infrastructure.^6^ More recently, a separate HRS study published in 2022 found that rural residence was associated with lower cognitive function, which was measured based on performance in word recall, serial subtraction of sevens and backwards-counting testing.^7^ Likewise, a 2023 study using a national US sample in the Reasons for Geographic and Racial Differences in Stroke Study data also found that rural residents had 34% significantly higher odds of incident cognitive impairment after adjusting for sociodemographic factors.^8^ The association between rural environments and the development of dementia may also be explained by the decreased opportunity for social interactions that exist in rural areas when compared with urban. Several studies have identified a link between decreased social interaction and cognitive decline,^9,10^ suggesting this as a potential underlying factor for these rural–urban disparities.

## Education and cognition

Based on recent data examining the relationship between educational attainment and cognition, low education status has been hypothesised as an additional risk factor for cognitive impairment. One 2024 study using a US sample of middle-aged and older adults from the 2021 Behavioral Risk Factor Surveillance System (BRFSS) found that, compared with respondents with less than a high school degree, some college education and a college degree were associated with 55 and 54% significantly lower odds of SCD, respectively, after adjusting for sociodemographic and health factors.^11^ Another 2024 study, using a 10-year prospective national US sample of older adults, found that, compared with those with less than a high school degree, obtaining a high school degree was associated with 29% significantly lower dementia risk; obtaining a college degree was further associated with a 43% significantly lower dementia risk after adjusting for sociodemographics and health.^12^ One theory that has been hypothesised to explain this protective effect of education on cognitive decline is related to the concept of cognitive reserve, which is defined as the ability of the brain to maintain cognition in response to ageing or disease.^13^ Higher educational background is widely considered to be a potential factor associated with improved cognitive reserve, and has therefore been theorised to be protective against cognitive decline.^13^

## Innovation and research aim

Although there has been research linking rural–urban residence and education to cognitive decline, to our knowledge there is no existing research that has examined the intersection among these three constructs. Therefore, our present study has two objectives: (a) to examine the association between rural–urban residence and SCD among US middle-aged and older adults; and (b) to examine whether education level moderates the association between rural–urban residence and SCD. Building on prior research, we hypothesised that residing in rural settings would be associated with increased odds of SCD. With this assumption, we also hypothesised that higher educational attainment would be more beneficial for rural residents, and would thus attenuate the probability of SCD compared with urban residents.

## Method

### Data source

Cross-sectional data were retrieved from the 2022 BRFSS through telephone surveys on health behaviours, conditions and preventive services. The BRFSS cognitive decline module was eligible only for adults of at least age 45 years, and this optional module was administered to 12 US states (FL, ID, IN, ME, NV, OR, RI, SC, UT, VA, VT and WI). The sample size was 63 890 respondents who completed this module and met the minimum age inclusion criteria.

The authors assert that all procedures contributing to this work comply with the ethical standards of the relevant national and institutional committees on human experimentation, and with the Helsinki Declaration of 1975 as revised in 2013. All procedures involving human subjects/patients were approved by the SUNY Upstate Institutional Review Board for the Protection of Human Subjects (no. 2221941-1). Verbal consent was witnessed and formally recorded. The BRFSS interviewers obtained verbal informed consent from all respondents during the initial contact and screening process, preceding all survey questions.

### SCD

SCD was the dependent variable. In the BRFSS cognitive decline module, respondents were asked, ‘During the past 12 months, have you experienced confusion or memory loss that is happening more often or is getting worse?’. Responses were binary (no or yes) and other responses (don’t know/not sure or refused) were coded as missing. A prior study has reported this BRFSS SCD variable to have high specificity and adequate sensitivity in psychometric testing.^14^

### Rural–urban residence

Rural–urban residence is a BRFSS-calculated binary variable in which respondents were categorised as residing in either a rural or urban county. This variable was developed using the National Center for Health Statistics Urban–Rural Classification Scheme for Counties, which uses the US Census as the underlying data. All counties are classified based on: (a) their status under the Office of Management and Budget designation as metropolitan, micropolitan or non-core, (b) the population size of metropolitan statistical areas (MSAs) and (c) the location of principal city populations within the largest MSAs. Rural was defined as non-metropolitan counties that did not qualify as micropolitan, and rural counties had a median population size of 11 218 people and population density of 18 persons per square mile. The BRFSS classified all other counties as urban.

### Education level

The highest level of education was a BRFSS-calculated variable in which respondents were classified into four categories: (a) less than high school (did not graduate), (b) graduated from high school, (c) some college (attended 1–3 years of college or technical school) and (d) graduated from college (at least 4 years).

### Covariates

Several sociodemographic and health variables were included as covariates in all logistic regression analyses. Sociodemographic covariates included age group (45–49, 50–54, 55–59, 60–64, 65–69, 70–74, 75–79 or 80+ years), sex (male or female), marital status (married or not married) and race and ethnicity (non-Hispanic White (hereafter, White), non-Hispanic Black (hereafter, Black), Hispanic, non-Hispanic Asian (hereafter, Asian) or non-Hispanic Other (hereafter, Other)). Health covariates included self-rated general health (poor, fair, good, very good or excellent), body mass index (BMI) category (underweight, normal weight, overweight or obese), cardiovascular disease (CVD) history, diabetes history, stroke history and depression history.

### Analysis plan

A Pearson chi-square test was conducted to examine the unadjusted association between rural–urban residence and SCD. We constructed three logistic regression models that sequentially adjusted for sociodemographic and health covariates. Model A was an unadjusted crude model containing only rural–urban residence, and model B was adjusted for all sociodemographic covariates. Our fully adjusted final model, model C, adjusted for all sociodemographic and health covariates. Our final logistic regression model had an average variance inflation factor of 1.11, which indicated that no multicollinearity was present. For our interaction model, we regressed SCD on the interaction between rural–urban residence and education, adjusting for all sociodemographic and health covariates. Finally, we also performed a sensitivity analysis in which we examined the rural–SCD association, along with the rural–education interaction on SCD, stratified by each of the eight age groups to account for differential life experiences.

Approximately 9% of the data was lost from listwise deletion and we did not identify any missing data patterns. The variable with the most missingness was BMI category (6.5%) followed by CVD history (1.2%). To maximise the full number of eligible respondents in the data and minimise bias from missing data, we utilised multiple imputation by chained equations (MICE) to generate 15 imputed data files with 10 iterations each for logistic regression analyses. Results from the MICE models are reported, but the conclusions were the same from listwise deletion. All analyses were conducted using Stata 18 with two-tailed tests at a 0.05 significance level.

## Results

### Sample characteristics

Among 63 890 US middle-aged and older adults in the BRFSS, about 81% were urban and 19% resided in rural geographic areas (Table 1). About 11% reported SCD, which was significantly more common among rural (12.0%) compared with urban (10.7%) residents (*X* ^2^[1] = 15.5, *P* < 0.001). Regardless of rural–urban residence, most respondents completed college (43.7%) and were in the 65–69 years age group (15.6%). Slightly more than half identified as female (55.4%) and married (57%). The sample was predominantly White (86.9%), followed by lower proportions of Black (6.6.%), Hispanic (3.6%) and Asian (0.7%). For health-related characteristics, most reported self-rated health as ‘very good’ (34.1%) and had a BMI overweight (36.8%). History of chronic and mental health conditions was fairly infrequent, with the most common being depression (19.4%) and diabetes (17.8%).

**Table 1:** Sample characteristics

Characteristics	Rural–urban residence		
	Urban	Rural	Total
	*n* = 51,711 (80.9%)	*n* = 12,179 (19.1%)	*N* = 63,890 (100%)
SCD	5539 (10.7%)	1456 (12.0%)	6995 (10.9%)
Highest education level			
<High school	2419 (4.7%)	630 (5.2%)	3049 (4.8%)
High school	11 638 (22.6%)	3461 (28.5%)	15 099 (23.7%)
Some college	14 233 (27.6%)	3463 (28.5%)	17 696 (27.8%)
College	23 204 (45.1%)	4582 (37.8%)	27 786 (43.7%)
Age group (years)			
45–49	4459 (8.6%)	819 (6.7%)	5278 (8.3%)
50–54	5928 (11.5%)	1143 (9.4%)	7071 (11.1%)
55–59	6143 (11.9%)	1391 (11.4%)	7534 (11.8%)
60–64	7402 (14.3%)	1809 (14.9%)	9211 (14.4%)
65–69	7910 (15.3%)	2060 (16.9%)	9970 (15.6%)
70–74	7735 (15.0%)	1945 (16.0%)	9680 (15.2%)
75–79	5860 (11.3%)	1504 (12.3%)	7364 (11.5%)
80+	6274 (12.1%)	1508 (12.4%)	7782 (12.2%)
Female	28 587 (55.3%)	6789 (55.7%)	35 376 (55.4%)
Married	29 349 (57.2%)	6817 (56.3%)	36 166 (57.0%)
Race and ethnicity			
White, non-Hispanic	44 386 (85.8%)	11 150 (91.6%)	55 536 (86.9%)
Black, non-Hispanic	3724 (7.2%)	494 (4.1%)	4218 (6.6%)
Hispanic	2064 (4.0%)	214 (1.8%)	2278 (3.6%)
Asian, non-Hispanic	411 (0.8%)	26 (0.2%)	437 (0.7%)
Other, non-Hispanic	1126 (2.2%)	295 (2.4%)	1421 (2.2%)
Self-rated health			
Poor	2586 (5.0%)	704 (5.8%)	3290 (5.2%)
Fair	7299 (14.2%)	1856 (15.3%)	9155 (14.4%)
Good	16 394 (31.8%)	3936 (32.4%)	20 330 (31.9%)
Very good	17 710 (34.3%)	4020 (33.1%)	21 730 (34.1%)
Excellent	7588 (14.7%)	1627 (13.4%)	9215 (14.5%)
BMI category			
Underweight	697 (1.4%)	164 (1.4%)	861 (1.4%)
Normal weight	13 522 (28.0%)	3 122 (27.4%)	16 644 (27.9%)
Overweight	17 922 (37.1%)	4079 (35.8%)	22 001 (36.8%)
Obese	16 194 (33.5%)	4032 (35.4%)	20 226 (33.9%)
Cardiovascular disease history	6314 (12.4%)	1679 (14.0%)	7993 (12.7%)
Diabetes history	9232 (17.9%)	2101 (17.3%)	11 333 (17.8%)
Stroke history	2972 (5.8%)	828 (6.8%)	3800 (6.0%)
Depression history	9970 (19.4%)	2354 (19.4%)	12 324 (19.4%)

SCD, subjective cognitive decline; BMI, body mass index.

### Main effects

In our unadjusted logistic regression model (model A), rural residence was associated with 13% significantly higher odds of SCD compared with urban residents (odds ratio 1.13, 95% CI 1.06–1.20, *P* < 0.001; Table 2). This association remained significant after adjusting for sociodemographic covariates in model B (adjusted odds ratio (aOR) 1.10, 95% CI 1.03–1.17, *P* < 0.01). Our fully adjusted model (model C), with all sociodemographic and health covariates indicated residing in rural areas, was associated with about 9% increased odds of SCD (aOR 1.09, 95% CI 1.02–1.16, *P* = 0.01).

**Table 2:** Adjusted odds of subjective cognitive decline, by rural–urban residence

Characteristic	Model A	Model B	Model C
	odds ratio (95% CI), *P*	aOR (95% CI), *P*	aOR (95% CI), *P*
Rural–urban residence			
Urban	Reference	Reference	Reference
Rural	1.13 (1.06–1.20), <0.001	1.10 (1.03–1.17), <0.01	1.09 (1.02–1.16), 0.01
Highest education level			
<High school		Reference	Reference
High school		0.72 (0.65–0.80), <0.001	0.94 (0.84–1.05), 0.26
Some college		0.69 (0.62–0.77), <0.001	0.94 (0.84–1.05), 0.29
College		0.49 (0.44–0.55), <0.001	0.85 (0.76–0.96), 0.01
Age group (years)			
45–49		Reference	Reference
50–54		1.06 (0.94–1.19), 0.37	1.03 (0.91–1.17), 0.64
55–59		1.12 (1.00–1.26), 0.06	1.06 (0.93–1.20), 0.39
60–64		1.07 (0.96–1.20), 0.23	1.00 (0.89–1.13), 0.97
65–69		0.99 (0.89–1.11), 0.91	0.98 (0.87–1.11), 0.76
70–74		1.09 (0.97–1.22), 0.15	1.04 (0.92–1.17), 0.55
75–79		1.21 (1.08–1.37), <0.01	1.25 (1.10–1.42), <0.01
80+		1.56 (1.39–1.74), <0.001	1.75 (1.55–1.98), <0.001
Female		0.92 (0.87–0.96), <0.01	0.83 (0.79–0.88), <0.001
Married		0.68 (0.65–0.72), <0.001	0.88 (0.83–0.93), <0.001
Race and ethnicity			
White, non-Hispanic		Reference	Reference
Black, non-Hispanic		0.93 (0.84–1.03), 0.19	0.94 (0.85–1.05), 0.27
Hispanic		1.18 (1.04–1.34), 0.01	1.17 (1.02–1.34), 0.02
Asian, non-Hispanic		1.03 (0.74–1.43), 0.86	1.12 (0.80–1.59), 0.50
Other, non-Hispanic		1.46 (1.26–1.69), <0.001	1.15 (0.98–1.34), 0.09
Self-rated health			
Poor			Reference
Fair			0.61 (0.56–0.67), <0.001
Good			0.33 (0.30–0.36), <0.001
Very good			0.19 (0.18–0.22), <0.001
Excellent			0.13 (0.11–0.15), <0.001
BMI category			
Underweight			0.93 (0.75–1.16), 0.51
Normal weight			Reference
Overweight			0.88 (0.82–0.94), <0.001
Obese			0.84 (0.78–0.90), <0.001
CVD history			1.17 (1.09–1.26), <0.001
Diabetes history			1.09 (1.02–1.16), 0.01
Stroke history			1.49 (1.37–1.63), <0.001
Depression history			3.22 (3.04–3.42), <0.001

aOR, adjusted odds ratio; CI, confidence interval; BMI, body mass index; CVD, cardiovascular disease.

Adjusted for sociodemographics (model B), increasingly higher levels of education were also significantly associated with lower odds of SCD. For instance, compared with those who did not complete high school, high school degree and college degree were associated with 28% (aOR 0.72, 95% CI 0.65–0.80, *P* < 0.001) and 51% (aOR 0.49, 95% CI 0.44–0.55, *P* < 0.001) lower odds of SCD, respectively. In the fully adjusted model, only a college degree was significantly associated with 15% lower odds of SCD (aOR 0.85, 95% CI 0.76–0.96, *P* = 0.01).

### Interaction effect

**Fig. 1:**
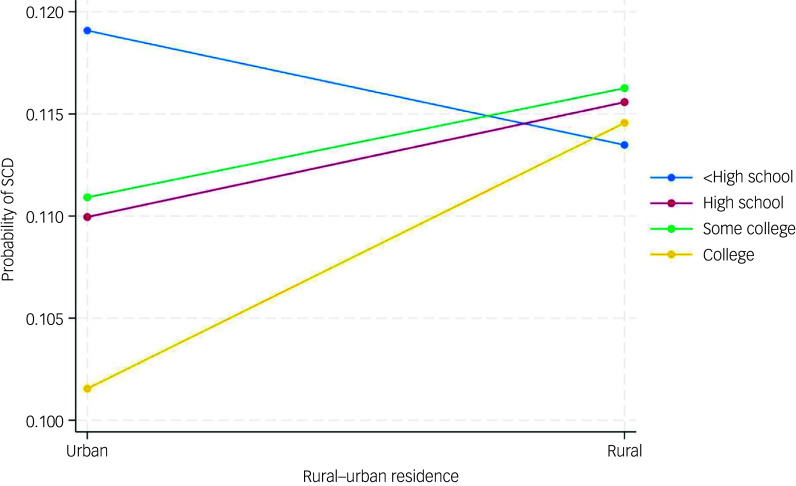
Moderating role of education level on association between rural–urban residence and SCD (subjective cognitive decline).

An interaction between rural–urban residence and education level was included in our fully adjusted logistic regression model. A statistically significant Wald test evaluated the joint significance of the interaction between rural–urban residence and education (*X* ^2^[6] = 13.5, *P* = 0.04). In particular, urban residents with increasingly higher levels of education had lower predicted probabilities of SCD (Fig. 1). For example, the predicted probability of SCD for college-educated urban residents was 10.2% (95% CI 9.7–10.6%) compared with 11.9% (95% CI 10.8–13.0%) for urban residents who did not complete high school. This contrast between the two educational levels among urban residents had a difference of 1.7%, which was statistically significant (95% CI 0.1–3.0%, *P* < 0.01). These educational benefits were not observed for rural residents, such as the predicted probability of 11.5% (95% CI 10.5–12.4%) for college graduates versus 11.3% (95% CI 9.2–13.5%) for those who did not complete high school, with the difference of 0.2% being non-significant (95% CI −2.2 to –2.5%, *P* = 0.93).

### Sensitivity analysis

We performed a sensitivity analysis examining the association between rural–urban residence and SCD, stratified by the eight age groups (Supplementary Table 1). In general, rural residence was associated with higher odds of SCD, although this relationship was significant for only three age groups throughout the 45–80+ years age range (50–54, 65–69 and 80+ years). For the urban–education interaction, higher levels of education did not have significant associations with SCD for generally the oldest age groups (55–59, 70–74, 75–79 and 80+ years).

## Discussion

This study analysed the association between rural–urban residence and SCD, and whether education may moderate this relationship. Our results indicate that, compared with urban settings, residing in rural areas was associated with significantly higher odds of SCD among US middle-aged and older adults. Higher levels of education were associated with significantly lower odds of SCD, and we found the probabilities of SCD to be lower only among urban residents who had obtained higher levels of education.

Our finding on the association between rurality and cognitive decline is consistent with numerous recent literature surrounding this topic. For example, a 2023 study identified a positive association between rural residence during childhood and poor cognition later in life.^15^ Similarly, a cross-sectional study conducted in 2021 identified significantly higher rates of dementia and mild cognitive impairment in rural individuals when compared with urban and suburban populations.^16^ While the results demonstrated in our study are aligned with these findings, the extent to which rurality plays a role in cognition remains unclear depending on the severity of cognitive impairment. For instance, one study reported an odds ratio of 3.76 when comparing the prevalence of mild cognitive impairment between rural and urban individuals.^17^ Another study identified a relative risk of dementia of about 79%,^6^ while our data in this present study suggested 9–13% increased odds for SCD for rural residents.

The results of our study also revealed an inverse association between education level and odds for cognitive decline, which aligns with much of the recent literature on this topic. Several studies have identified improvements in language, memory, attention and executive function in individuals who had attained higher education.^18–20^ Our data similarly suggested that individuals who had attained a college degree have a 15% decreased odds for SCD when compared with those who did not complete high school. Many studies theorise that cognitive reserve is the underlying mechanism that explains the improved cognition in older adults who have a greater educational background.^19,21,22^ Additionally, a 2019 study identified decreased decline of grey and white matter structure in patients who had attained higher levels of education,^18^ offering an additional potential mechanism for improved cognition later in life.

When comparing the influence of education on SCD in rural versus urban settings, our findings indicate that this has a limited benefit on SCD in rural environments while playing a greater role in urban populations, especially for those who are middle-aged compared with older adults. Given these findings, we theorise that education-based approaches aimed at preventing or slowing cognitive decline are more effective at earlier ages. This may be due to older adults often retiring from the workforce or decreased neuroplasticity at higher ages. Additional exploration is needed to identify the underlying mechanisms that contribute to differences in educational benefits between age groups. Our results also contradict one of our initial hypotheses that theorised an increased benefit of education on SCD in rural communities when compared with urban ones. One potential explanation for this result is based on the predominant occupation and lifestyle of rural versus urban residents. Occupation has been shown to play a role in health, with higher-paying, more stable jobs being associated with improved health outcomes.^23,24^ These optimal jobs are generally achieved through the increased opportunity for upward mobility provided by higher education.^25^ This upward mobility, however, is not consistent between urban and rural communities. Generally, there is more opportunity for high-paying jobs for urban residents when compared with rural ones.^26^ Similarly, the occupations in these two communities are also different, with urban residents being more likely to be employed in cognitively demanding jobs with rural residents being more likely to be employed in demanding physical occupations.^27^ We theorise that the limited opportunities for higher-paying jobs in rural communities signifies that higher education is less consequential for occupation status and therefore plays a decreased role in cognitive health.

Another potential factor that may explain our findings is the disparity in healthcare that exists between rural and urban environments. The difference in access to healthcare between urban and rural areas is well described in the literature, with several studies identifying poor outcomes in several key areas, such as mortality rate and life expectancy, for rural residents when compared with their urban counterparts.^28–30^ This disparity of health outcomes is prevalent for degenerative cognitive disease, as demonstrated in recent articles.^6,16,31,32^ It is possible that, in urban areas, individuals who are highly educated are more able to exercise their health literacy and navigate the available health systems. This may allow these urban residents to utilise the health systems more appropriately in order to improve and maintain their cognitive health.^33^ Likewise, we theorise that limited access to preventative medicine and mental healthcare in rural settings may reduce the potential benefits of education on cognitive health.^34^ This, therefore, is a potential driving factor that may underlie the differential effect of higher education on the odds of SCD when comparing urban and rural populations.

The results of our study have implications for research, clinical practice and policy in regard to improved outcomes for SCD. In terms of research, additional studies are required to better understand the mechanisms that drive SCD. In particular, it is important to identify the specific pathways that define the association between education and SCD, especially characterisation of the factors that may allow education to improve cognition in urban populations, but not rural ones. Clinically, these data highlight several potential key risk factors for degenerative cognitive disease, which can guide clinical decision-making. This study identified both low education level and rural residence as potential risk factors for SCD, which in turn can be an indicator of neurocognitive disease. A patient who presents with these risk factors may require closer surveillance when screening for potential cognitive disorder. With regard to policy, improvements in and access to higher education are vital for improving outcomes of SCD; affordable and accessible education may help mitigate SCD. Although our results indicate a statistically significant association, additional analyses are needed to identify whether these educational benefits may translate to meaningful clinical significance and economic gain for the healthcare system. Nonetheless, our findings suggest that improving rates of higher education, particularly in urban areas, may have a positive impact on the odds of SCD and future cognitive decline.

There are several limitations to our study. First, the annual BRFSS data are cross-sectional, which inhibits causal associations between rurality and cognition. The cross-sectional nature of our data set introduces a limitation with regard to longitudinal changes in cognition throughout life. Individuals with different levels of initial cognition early in life may demonstrate varying transitions of cognition over time, and self-selection into higher education or rural–urban preferences, impacting their risk of SCD. Second, our measure for cognitive decline is subjective. It is possible that perceived cognitive impairment may differ depending on education and, more specifically, on health education. Third, geographic data are limited in the BRFSS, which allowed for only two classifications as either rural or urban. Finally, due to a large amount of missing data, our analyses were unable to adjust for socioeconomic factors such as income and assets, which is important to consider because these may potentially confound the association between rural–urban residence and SCD. Despite these limitations, the BRFSS is the ideal data-set for this investigation because rural–urban residence and cognition data are publicly available for a large national US sample. In addition, our present investigation is noteworthy because we believe it is among the first studies to analyse the interplay between rurality, education and cognition. Further research is needed to examine whether these associations are observed with other demographic groups, such as across different sex and racial-ethnic groups.

This study demonstrates the associations of education level and rural–urban residence with SCD. Our findings suggest that lower level of education and rural residence are both associated with higher odds of SCD. The data also reveal that increasingly higher levels of education are a protective factor against SCD in urban populations, but do not play a significant role in SCD for rural communities. These results identify key areas of improvement: increased access to education, updated screening guidelines for individuals at risk of degenerative cognitive disease and further research into other factors that underlie the development of SCD and impaired cognition.

## Supporting information

Wong and Mansour supplementary material Wong and Mansour supplementary material

## Data Availability

This study uses public BRFSS data, which may be obtained through the United States Centers for Disease Control and Prevention website: https://www.cdc.gov/brfss/index.html

## Supplementary material

The supplementary material is available online at https://doi.org/10.1192/bjo.2025.45
