# Supplementary material for: Diverging cognitive benefits from education between rural and urban middle-aged and older adults in the USA
Source: BJPsych Open. 2025 Apr 17;11(3):e88. doi: 10.1192/bjo.2025.45 (PMC12052571; doi:10.1192/bjo.2025.45)
Supplement: Wong and Mansour supplementary material [file S2056472425000456sup001.docx]

# **Supplemental Table 1. Adjusted Odds of Association Between Rural Residence and Subjective Cognitive Decline, Stratified by Age Group**

| Age Group | Adjusted Odds Ratio (95% CI), p |
| --- | --- |
| 45-49 | 1.02 (0.78-1.34), .86 |
| 50-54 | 1.25 (1.01-1.56), .04 |
| 55-59 | 1.09 (0.89-1.33), .42 |
| 60-64 | 0.88 (0.73-1.06), .18 |
| 65-69 | 1.25 (1.06-1.48), <.01 |
| 70-74 | 0.96 (0.81-1.14), .63 |
| 75-79 | 1.05 (0.87-1.26), .60 |
| 80+ | 1.20 (1.03-1.40), .02 |

*Note:* Urban is the reference group for rural-urban residence. Logistic regression model is adjusted for highest education level, sex, marital status, race and ethnicity, self-rated general health, body mass index category, cardiovascular disease history, diabetes history, stroke history and depression history.
